# A Comprehensive Systematic Study on Thermoresponsive Gels: Beyond the Common Architectures of Linear Terpolymers

**DOI:** 10.3390/polym9010031

**Published:** 2017-01-20

**Authors:** Anna P. Constantinou, Hanyi Zhao, Catriona M. McGilvery, Alexandra E. Porter, Theoni K. Georgiou

**Affiliations:** Department of Materials, Imperial College London, Royal School of Mines, Exhibition Road, London SW7 2AZ, UK; anna.constantinou14@imperial.ac.uk (A.P.C.); hanyi.zhao15@imperial.ac.uk (H.Z.); catriona.mcgilvery@imperial.ac.uk (C.M.M.); a.porter@imperial.ac.uk (A.E.P.)

**Keywords:** thermoresponsive polymers, 2-(dimethylamino)ethyl methacrylate, injectable gels, 3-D printing, group transfer polymerization (GTP), terpolymers, complex architectures, well-defined polymers

## Abstract

In this study, seven thermoresponsive methacrylate terpolymers with the same molar mass (MM) and composition but various architectures were successfully synthesized using group transfer polymerization (GTP). These terpolymers were based on tri(ethylene glycol) methyl ether methacrylate (TEGMA, A unit), *n*-butyl methacrylate (BuMA, B unit), and 2-(dimethylamino)ethyl methacrylate (DMAEMA, C unit). Along with the more common ABC, ACB, BAC, and statistical architectures, three diblock terpolymers were also synthesized and investigated for the first time, namely (AB)C, A(BC), and B(AC); where the units in the brackets are randomly copolymerized. Two BC diblock copolymers were also synthesized for comparison. Their hydrodynamic diameters and their effective p*K*_a_s were determined by dynamic light scattering (DLS) and hydrogen ion titrations, respectively. The self-assembly behavior of the copolymers was also visualized by transmission electron microscopy (TEM). Both dilute and concentrated aqueous copolymer solutions were extensively studied by visual tests and their cloud points (CP) and gel points were determined. It is proven that the aqueous solution properties of the copolymers, with specific interest in their thermoresponsive properties, are influenced by the architecture, with the ABC and A(BC) ones to show clear sol-gel transition.

## 1. Introduction

Thermoresponsive polymers are “smart” polymers which are able to respond to temperature [1,2,3]. The response of the polymers is indicated by a change of their properties. A special class of thermoresponsive polymers which has become very popular covers polymers that exhibit a Lower Critical Solution Temperature (LCST) behavior. These polymers become insoluble in aqueous media when increasing the temperature. This behavior is explained by the “hydrophobic effect”, i.e., the entropy of water becomes the most dominant factor and forces the polymer to precipitate out of solution [2,4]. It is the same phenomenon that in lower temperatures and concentrations forces the polymers to form micelles [2,4]. Thermoresponsive polymers with the appropriate structural parameters, and under the appropriate environmental conditions form 3-D networks of physically-interconnected micelles; these 3-D networks are known as thermoresponsive gels [5]. As has been well-demonstrated, the architecture, the molar mass (MM), the composition, and the molar mass distribution (MMD) of the thermoresponsive copolymers determine whether, and at which temperature and concentration a gel is formed [1]. In the case of aqueous solutions, these micelles are connected via well-hydrated bridges [5].

Thermoresponsive gels have been widely studied on account of their interesting applications, including tissue engineering as injectable gels [6,7,8] and more recently in 3-D printing [9,10,11,12]. For thermoresponsive polymers to be used as injectable gels a sol-gel transition close to body temperature is required for a minimal invasion administration [13]. On the other hand, for the 3-D printing application the sol-gel transition should occur either close to room or body temperature, depending on the temperature that a stable printed structure is desirable at [11,14]. In this case, the thermoresponsive gel should not only possess good mechanical properties in order to maintain the printed structure, but it should also be characterized by good shear-thinning properties [11,14].

For thermoresponsive polymers to be widely applied and industrialized, their synthesis should be easy, time- and cost-effective, reproducible, and scalable. Furthermore, a “living” or “controlled” polymerization technique is required for the synthesis in order to produce copolymers with well-defined structural parameters [15] since it has been well-demonstrated in the literature that these parameters can affect the sol-gel transition [1]. Group transfer polymerization (GTP) is an ideal polymerization method for the synthesis of methacrylate well-defined polymers because: (i) it is a fast polymerization technique (10–15 min per block) [16]; (ii) it produces polymers with narrow MMD (usually below 1.2) and well-defined and controllable composition [16,17]; (iii) it can produce polymers in an industrial scale and (iv) it is cost-effective [17]. GTP is cost-effective for several reasons: it works at higher concentrations than for example anionic polymerization, it is performed at room temperature so there is no need to cool down or heat up the reaction, and there is a 100% conversion of the monomer to the polymer so sequential one-pot polymerization can be easily achieved for block copolymers without any extra pot(s) or purification steps needed [17]. Therefore, this polymerization technique has been used in the present study and the aim was to investigate how the polymers’ architecture affects their polymers’ thermoresponsive behavior.

Our group has previously published five research articles on thermoresponsive gels in which several polymeric parameters have been varied [18,19,20,21,22]. In four of the studies, the effects of architecture [18,20], composition [20,21,22], and MM [21] of triblock and statistical terpolymers on the thermoresponsive properties have been systematically investigated. These terpolymers were based on (i) a poly(ethylene glycol) (PEG) methacrylate unit as the hydrophilic and biocompatible unit (A unit); (ii) an alkyl methacrylate hydrophobic unit (B unit); and (iii) the thermoresponsive and pH-responsive 2-(dimethylamino)ethyl methacrylate (DMAEMA, C unit). The lengths of the alkyl and the PEG side-chains have been also systematically varied by using: (i) ethyl, *n*-butyl, and *n*-hexyl methacrylate (EtMA, BuMA, and HexMA, respectively) [18]; and (ii) di-, penta-, and nona(ethylene glycol) methyl ether methacrylate (DEGMA, PEGMA, and NEGMA, respectively) [22]. It has been demonstrated that the optimum parameters producing polymers with the clearest sol-gel transition and gels of good mechanical properties are: (i) the ABC architecture [18,20]; (ii) MM ranging between 7000–10,000 g·mol^−1^ [21]; (iii) intermediate hydrophobic composition of around 30%–35% *w*/*w* [20,21,22]; (iv) BuMA as the hydrophobic unit [18]; and (v) a PEG methacrylate unit with ethylene glycol groups between two and five [22].

Given these optimum parameters, new copolymers which are based on the unique combination of these structural characteristics were designed. Therefore, the copolymers are based on BuMA, and DMAEMA as B and C units, respectively, while the A unit was based on tri(ethylene glycol) methyl ether methacrylate (TEGMA). We chose TEGMA instead of the 300 g·mol^−1^ PEGMA that we normally use because based on our latest study we think it will improve the thermoresponsive ability of the terpolymers. The target TEGMA-BuMA-DMAEMA composition was 25%–35%–40% *w*/*w*, while the target MM was 8200 g·mol^−1^. Both the composition and the MM values are within the intermediate range established previously. The aim of this study is not only to use the optimum structural parameters, but also to examine interesting architectures of terpolymers which have not been systematically investigated before. More specifically, apart from the ABC, ACB, BAC, and statistical architectures that have been previously studied, in the present study diblock terpolymers have also been synthesized. The three possible combinations that produce different diblock terpolymers are: (i) (AB)C, A(BC), and B(AC); the units in the brackets have been randomly copolymerized and the resulting block has been polymerized with the third monomer to form the final diblock terpolymer. Two BC diblock bipolymers have also been synthesized to mimic: (i) the BuMA:DMAEMA weight percentages in the terpolymers, and (ii) the hydrophobic:hydrophilic [BuMA:(DMAEMA + TEGMA)] weight percentages in the terpolymers. To the best of our knowledge this is the first time so many different architectures have been synthesized and investigated in terms of their thermoresponsive behavior. Furthermore, it should be noted that the architecture was able to be varied independently i.e., without altering the MM and the composition that is not easy to achieve, but it was because GTP was used.

## 2. Experimental

### 2.1. Materials

TEGMA (monomer, MM = 232.27 g·mol^−1^, 94%), BuMA (monomer, 99%), DMAEMA (monomer, 98%), activated basic aluminum oxide (Al_2_O_3_·KOH), calcium hydride (CaH_2_, ≥90%), 2,2-diphenyl-1-picrylhydrazyl (DPPH, free-radical inhibitor), potassium metal, sodium metal, tetrahydrofuran (THF, polymerization solvent, HPCL grade, ≥99.9%), methyl trimethylsilyl dimethylketene acetal (MTS, initiator, 95%), deuterated chloroform (chloroform-d, 99.8 atom% D), sodium hydroxide pellets (NaOH, 97%), concentrated hydrochloric acid (HCl, ACS reagent, 37%), and hydrochloric acid solution (volumetric, 1M) were purchased from Sigma Aldrich Co Ltd., Irvine, UK. Tetrabutylammonium hydroxide (40% in water) and benzoic acid were purchased from Acros Organics—UK distributor Fisher Scientific UK Ltd., Loughborough, UK. Tetrahydrofuran (THF, mobile phase in GPC, GPC grade) and *n*-hexane (precipitation solvent) were purchased from Fisher Scientific UK Ltd. (Loughborough, UK) and VWR International Ltd. (Lutterworth, UK), respectively. Phosphate buffered saline (PBS, 10x solution) was purchased from Fischer Scientific UK Ltd., Loughborough, UK.

### 2.2. Purification of the Starting Materials

**Figure 1:**
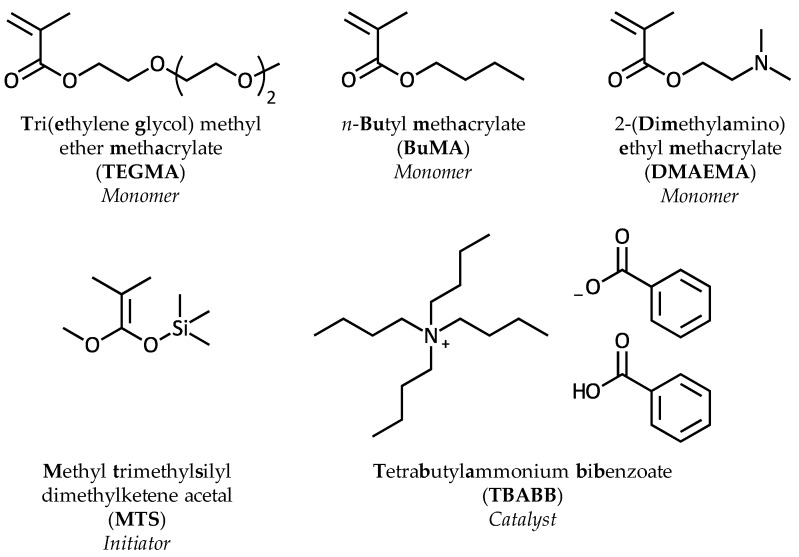
Chemical structures of the monomers, the initiator, and the catalyst.

The monomers, TEGMA, BuMA, and DMAEMA, were passed twice through Al_2_O_3_·KOH in order to remove the inhibitor (monomethyl ether hydroquinone) and any acidic impurities. DPPH was added in order to prevent free-radical polymerization and the humidity was eliminated by stirring the monomers over CaH_2_ for 3 h. The monomers were then kept refrigerated until use. The polymerization solvent, THF, was dried by refluxing for 3 days over potassium and sodium metals. The initiator, MTS, and the monomers were distilled under vacuum prior to polymerization. The catalyst, tetrabutylammonium bibenzoate (TBABB), was previously synthesized by tetrabutylammonium hydroxide and benzoic acid, as reported by Dicker et al. [23], and it was dried and kept under vacuum until use. All the glassware was dried overnight at 140 °C and assembled hot under vacuum. The chemical structures of the monomers, the initiator and the catalyst are shown in Figure 1.

### 2.3. Copolymer Synthesis

All the copolymers were synthesized using a cost-effective and easy to scale-up anionic polymerization technique, specifically group transfer polymerization (GTP), which enables the synthesis of each block in only 10–15 min. The block copolymers were synthesized via sequential addition of the monomers. As an example, the synthesis of polymer 1, namely TEGMA_9_-*b*-BuMA_20_-*b*-DMAEMA_21_ follows: As a first step, TBABB (~10 mg) was added in a 250 mL round-bottom flask. This flask was sealed with a rubber septum and purged with argon, followed by the addition of 60 mL freshly-distilled THF using a syringe. MTS (0.37 mL, 0.32 g, 1.8 mmol) was then syringed into the flask. The addition of the TEGMA monomer (3.6 mL, 3.7 g, 15.9 mmol) followed and the exothermic reaction was monitored; the temperature was increased from 24.2 to 28.3 °C. As soon as the reaction was complete, two samples of 0.1 mL were obtained for GPC and ^1^H-NMR analysis. Following this, BuMA (5.8 mL, 5.2 g, 36.3 mmol) was added, thus the temperature was increased from 25.2 to 28.9 °C. Two samples of 0.1 mL were obtained in order to analyze them by GPC and ^1^H-NMR. As a last step, 6.3 mL of DMAEMA (5.9 g, 37.5 mmol) was added and an exotherm from 27.6 to 31.8 °C was observed. GPC and ^1^H-NMR samples (0.1 mL each) were extracted for analysis. The polymers were precipitated in cool *n*-hexane and dried in a vacuum oven at room temperature. In this study, seven terpolymers of the same composition and target MM, but different architectures were synthesized. This was achieved by keeping the amount of monomers the same, while the order of each monomer addition was varied. More specifically, concerning the diblock terpolymers, the statistical bipolymer was synthesized via simultaneous addition of the two monomers. The statistical terpolymer was synthesized by adding all the monomers prior to the addition of the MTS. Two diblock copolymers based only on BuMA and DMAEMA with different compositions were also synthesized by adding different amounts of the monomers.

### 2.4. Characterization in Organic Solvents

The MM, the molar mass distribution (MMD), and the composition of all the copolymers and their linear precursors were determined in organic solvents.

#### 2.4.1. Gel Permeation Chromatography (GPC)

The final copolymers and their precursors were characterized in terms of their MM and MMD by GPC. For this, an Agilent SECurity GPC system, with a Polymer Standard Service (PSS) SDV analytical linear M column (SDA083005LIM) was used (Agilent technologies UK Ltd., Shropshire, UK). This system is equipped with a “1260 Iso” isocratic pump and an Agilent 1260 refractive index (RI) detector. As a mobile phase, THF with 5% vol triethylamine was used, which was pumped with a flow rate of 1 mL·min^−1^. The calibration curve was plotted by running six different linear poly(methyl methacrylate) (PMMA) standard samples with MM equal to 2000, 4000, 8000, 20,000, 50,000, and 100,000 g·mol^−1^, purchased from Fluka, Sigma Aldrich Co Ltd., Irvine, UK.

#### 2.4.2. Proton Nuclear Magnetic Resonance Spectroscopy (^1^H-NMR)

The final copolymers and their precursors were characterized in terms of their composition by ^1^H-NMR using a 400 MHz Avance Bruker NMR spectrometer (Bruker UK Ltd., Coventry, UK). The ^1^H-NMR spectra were obtained using CDCl_3_ as the deuterated solvent.

### 2.5. Characterization in Aqueous Solution

The effective dissociation constants (p*K*_a_), the hydrodynamic diameters (*d*_h_), the cloud points (CP), and the thermal response of copolymers in aqueous solutions were determined.

#### 2.5.1. Hydrogen Ion Titrations

Hydrogen ion titrations of 1% *w*/*w* aqueous polymer solutions were performed by using a HI98103 pH-checker from Hanna instruments Ltd., Leighton Buzzard, UK. The solutions were titrated from pH 2 to pH 12 using a 0.25 M NaOH solution. The start- and end-point of the titration of the DMAEMA units were determined by plotting the first derivative of the titration curve and the effective p*K*_a_ was determined as the pH of the solution at which the amino groups were protonated by 50%.

#### 2.5.2. Dynamic Light Scattering (DLS)

DLS measurements of 1% *w*/*w* aqueous polymer solutions (pH adjusted to 6 and 7) were conducted by using a Zetasizer Nano ZSP instrument from Malvern Instruments Ltd., Malvern, UK. The polymer solutions were filtered using nylon 0.45 μm PTFE syringe filters in order to remove any dust and bigger aggregates. After filtering, the solutions were allowed to settle in order to ensure complete bubble removal. Three DLS experiments were performed per sample at room temperature and the scattered light was collected at a backscatter angle of 173°. The *d*_h_s reported are the mean values determined as the diameters corresponding to the peak of maximum intensity.

These experimental *d*_h_s were compared to the theoretical ones. Four different theoretical models were applied depending on the polymer architecture. Concerning the block copolymers, formation of spherical micelles was assumed and the calculations were based on the projected length of the methacrylate unit, equal to 0.254 nm, and the experimental degree of polymerization (DP). (1) When the ABC and BC architectures are concerned, the theoretical diameter was calculated as follows: *d* = (DP_BuMA_ + 2 × DP_DMAEMA_) × 0.254 nm; (2) When the hydrophobic BuMA unit forms a distinct block at the end of the polymer chain, i.e., ACB, BAC, and B(AC), the theoretical values were calculated using the following equation: *d* = [DP_BuMA_ + 2 × (DP_DMAEMA_ + DP_TEGMA_)] × 0.254 nm; (3) When the hydrophobic BuMA unit is randomly copolymerized with either of the hydrophilic monomer (TEGMA or DMAEMA), while the other hydrophilic monomer is in a block structure, i.e., (AB)C and A(BC), the following formulae were used: *d* = (DP_BuMA_ + DP_TEGMA_ + 2 × DP_DMAEMA_) × 0.254 nm and *d* = (DP_BuMA_ + DP_DMAEMA_ + 2 × DP_TEGMA_) × 0.254 nm; (4) When a random copolymer is concerned, random coil is assumed to be formed, the diameter of which is calculated according to the following equation: <*d*_g_^2^>^1/2^ = 2 × [2 × 2.20 × (DP_TEGMA_ + DP_BuMA_ + DP_DMAEMA_)/3]^1/2^ × 0.154 nm. For these calculations, the experimental DPs were used, as calculated from GPC and ^1^H-NMR results.

#### 2.5.3. Transmission Electron Microscopy (TEM)

The TEM images were recorded using an FEI Titan 80–300 transmission electron microscope (TEM) (FEI (part of Thermo Fisher Scientific, Hillsboro, OR, USA), equipped with an image corrector. The instrument was operated at 80 kV to enhance contrast for bright field TEM, and an objective aperture of 70 µm was used. 1% *w*/*w* aqueous copolymer solutions (pH adjusted at 6) was used for the preparation of the TEM samples. The TEM samples were prepared by pipetting 3.5 μL of solution onto holey-carbon grids. After two minutes any excess of solvent was removed using filter paper. To increase contrast in the TEM the samples were then negatively stained by adding 30 μL of 1% *w*/*v* uranyl acetate solution to the grid, while the TEM grids were held at an angle of 45°. Any remaining excess solution was removed with filter paper, and the grids were left to dry.

#### 2.5.4. Visual Tests

The visual tests were performed using an IKA RCT stirrer hotplate (IKA^®^ England Ltd., Oxford, UK), equipped with an IKA ETS-D5 temperature controller, and a continuously-stirred water-bath. For the determination of the CPs, 1% *w*/*w* aqueous copolymer solutions were used, whereas for the construction of the phase diagrams, 1%, 2%, 5%, 10%, 15%, 20%, 25%, and 30% *w*/*w* copolymer solutions in PBS, were tested. In both cases, the DMAEMA units were protonated by 10% to enhance solubility. The vials were suspended in a water-bath and a thermal response was visually inspected every one degree from 20 to 80 °C. The CP was determined as the temperature at which the solution turned cloudy, while the gel point was determined as the temperature at which a stable gel was formed, which did not flow upon tube inversion.

## 3. Results and Discussion

**Figure 2:**
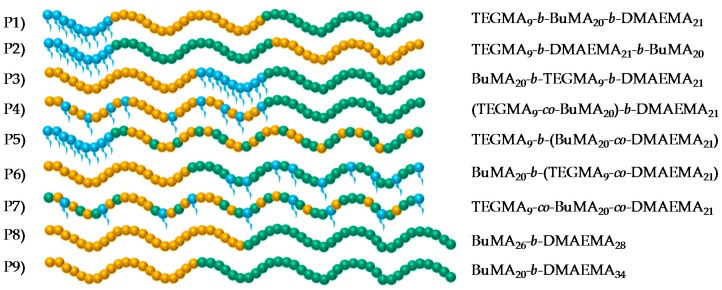
Schematic showing the structures of all the copolymers studied. Blue, orange, and green colors represent the TEGMA, BuMA, and DMAEMA units, respectively.

In this study, the synthesis of seven terpolymers was achieved using GTP and the architecture was systematically varied, while the TEGMA-BuMA-DMAEMA composition and the MM were kept constant at 25%–35%–40% *w*/*w* and 8200 g·mol^−1^, respectively. More specifically, three triblock terpolymers (ABC, CAB, and BAC), three diblock terpolymers ((AB)C, A(BC), and B(AC)) and one statistical copolymer were synthesized in order to investigate the effect of architecture on the thermoresponsive behavior. For comparison, two BC diblock copolymers were also synthesized, with the same MM but with the BuMA-DMAEMA weight percentages mimicking the BuMA-DMAEMA and the hydrophobic-hydrophilic weight ratio in the terpolymers. The structures of the copolymers are schematically shown in Figure 2. The TEGMA, BuMA, and DMAEMA units are represented by blue, orange, and green spheres, respectively.

### 3.1. Structural Properties

Table 1 summarizes the structural properties of all the copolymers and their linear precursors. Specifically, the theoretical MMs, and the experimental MMs and MMDs as resulted from GPC analysis are shown; the latter are shown as dispersity indices (*Ð*). The theoretical compositions and the experimental ones, as resulted by ^1^H-NMR analysis, are also listed in Table 1.

#### 3.1.1. Molar Masses and Molar Mass Distributions

As can be seen in Table 1, the number-average MM (*M*_n_) values of the final copolymers vary between 8550 and 11,000 g·mol^−1^, which are within the desirable range for obtaining a clear sol-gel transition. When the experimental *M*_n_ values of the final copolymers and their precursors are compared to the theoretical ones, they are slightly higher. This is ascribed to (i) the calibration curve being based on PMMA standard samples, and (ii) the partial deactivation of the initiator, MTS, i.e., an amount of the initiator molecules is terminated by the presence of humidity or any other protic impurities. This is consistent with other studies on polymers synthesized via GTP [18,19,20,21,22,24].

In Table 1, the *Ð* values are also given, which are satisfactorily close to the ideal value of unity (varying between 1.07 and 1.17). This confirms the successful “living” GTP, similar to previously reported studies [18,19,20,21,22,24]. Concerning the *Ð* values of the final copolymers, it can be generally observed that lower *Ð* values were obtained for the BC diblock copolymers; these copolymers are the BuMA_26_-*b*-DMAEMA_28_ (Polymer 8) and BuMA_20_-*b*-DMAEMA_34_ (Polymer 9). This can be attributed to the absence of the TEGMA units, which are macromonomers with average MM, thus meaning that they have wider MMD compared to the BuMA and DMAEMA monomers with well-defined structure and MM. This is supported by other GTP studies in which a PEGMA-based macromonomer has been incorporated into the polymer structure [22,25]. Also, lower *Ð* values are observed in the case of the statistical copolymer TEGMA_9_-*co*-BuMA_20_-*co*-DMAEMA_21_ (Polymer 7) and the (TEGMA_9_-*co*-BuMA_20_)-*b*-DMAEMA_21_ (Polymer 4). To produce the statistical copolymer, Polymer 7, all monomers were added to the flask prior to the initiator and the flask was cooled down in a room temperature water bath to avoid the flask overheating because the monomers were not added dropwise. This resulted in a more controlled polymerization and a synthesized polymer with a narrower MMD was produced unlike previous studies where the flask was not cooled down and the statistical copolymer had a broader *Ð* [19,22]. Polymer 4 (TEGMA_9_-*co*-BuMA_20_)-*b*-DMAEMA_21_ also had a very narrow MMD probably due to the first block being a statistical block that contained TEGMA and its simultaneous polymerization with BuMA assisted a controlled polymerization.

**Figure 3:**
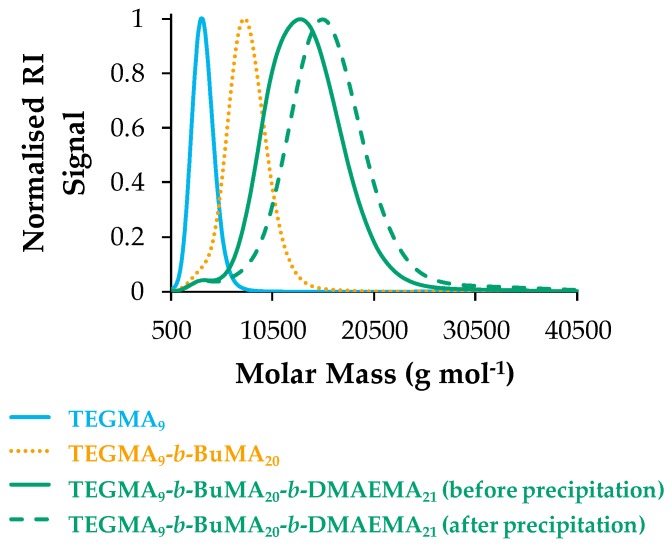
Gel permeation chromatography (GPC) traces of Polymer 1 (TEGMA_9_-*b*-BuMA_20_-*b*-DMAEMA_21_) and its precursors. The TEGMA homopolymer (TEGMA_9_), the diblock (TEGMA_9_-*b*-BuMA_20_), and the triblock copolymer before precipitation are shown in blue solid, orange dotted and green solid lines, respectively. The triblock copolymer after precipitation is indicated by green dashed line.

Figure 3 shows the GPC traces of the TEGMA_9_-*b*-BuMA_20_-*b*-DMAEMA_21_ (Polymer 1) before and after precipitation, shown in green solid and dashed line, respectively, and its precursors (TEGMA_9_ and TEGMA_9_-*b*-BuMA_20_, colored in blue and orange, respectively). Figure 3 confirms the successful sequential GTP since the peak appears at higher MM as the polymerization progresses (from the homopolymer to the diblock to the triblock). By precipitating, the lower polymer chains are lost in the precipitation solvent, thus the MM shifts at higher MM. The low intensity shoulder at lower MM corresponds to the TEGMA homopolymer, which slightly prevented further polymerization. Also, it is worth-noting that no peak related to the monomers was observed, thus indicating complete consumption and 100% conversion of the monomers to the polymer. These observations are the same for all the copolymers, the GPC traces of which can be found in Figure S1 in the Supplementary Materials.

#### 3.1.2. Compositions

The ^1^H-NMR results show good agreement between the theoretical and experimental compositions of the final copolymers and their precursors; these values are listed in Table 1. The experimental compositions were calculated by using the integral of three distinctive peaks belonging to the three different repeated units (see Figure S2 in the Supplementary Materials, which shows the NMR spectra of Polymer 1 and its precursors). The distinctive peak of the TEGMA unit is the one at 3.35 ppm and belongs to the three methoxy protons, whereas the one of BuMA appears at 3.9 ppm and belongs to the two methylene protons of the side-chain closest to the ester. The peak at 2.25 ppm is the one used for DMAEMA and it corresponds to the six methyl protons next to the nitrogen.

### 3.2. Aqueous Solution Properties

#### 3.2.1. Hydrodynamic Diameters

Table 2 lists the theoretical hydrodynamic diameters, calculated by assuming spherical micelle and random coil formation by the block and statistical copolymers, respectively. The experimental hydrodynamic diameters of the copolymers in aqueous solutions at both pH 6 and 7, as obtained by DLS, are also summarized in Table 2.

**Figure 4:**
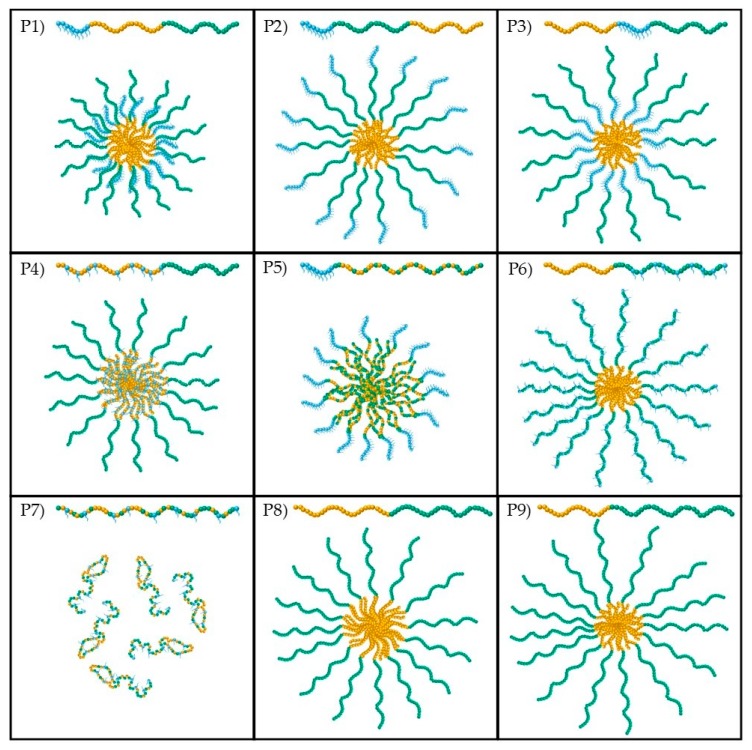
Schematic showing the structures of the spherical micelles adopted by the block copolymers (Polymers 1–6, 8, and 9). The random coil formed by the statistical copolymer (Polymer 7) is also shown. The TEGMA, BuMA, and DMAEMA units are colored in blue, orange, and green, respectively.

Concerning the theoretical calculations, the amphiphilic block copolymers are assumed to form spherical micelles with the BuMA-based block (either as homopolymer or random copolymer with one of the hydrophilic units) to form the core of the micelle. The structures of the spherical micelles, as well as the random coil configuration assumed to be adopted by the block and the statistical copolymers, respectively, are illustrated schematically in Figure 4. The TEGMA, BuMA, and DMAEMA units are represented by blue, orange, and green spheres, respectively.

As can be seen in Table 2, the experimental hydrodynamic diameters of the block terpolymers depend on the polymer architecture. At both pH values, the experimental sizes of Polymer 1, and 3, with ABC and BAC architecture, respectively, are smaller than the theoretical ones, as expected and observed before [20,21,22]. This is also valid for the solution of Polymer 6 (with B(AC) architecture) at pH 6, at which the DMAEMA units are more than 50% protonated (as it is discussed in the section on effective p*K*_a_). This trend can be attributed to the theoretical model assuming that (i) the methacrylate backbone is fully extended and (ii) the BuMA-based block fully overlaps. In reality, this is not the case since (i) the hydrophobic block is in the collapsed state on account of its incompatibility with the aqueous solvent and (ii) the polymer chains might overlap in a bigger extent, thus reducing the size of the micelles. The opposite effect is observed in the rest of the cases on block terpolymers, i.e., Polymer 2, 4, 5 with ACB, (AB)C, and A(BC) architecture at both pH values and Polymer 6 [B(AC)] at pH 7. This can be attributed to several factors including (i) the DP_BuMA_ being higher than in the other studies, or the difference in hydrophilicity between the repeated units being less pronounced, i.e., at pH 7, thus enhancing the hydrophobic interactions and aggregation, (ii) the core-forming block overlapping in a lesser extent, and (iii) the theoretical model of Polymer 4 and 5 assuming that the hydrophilic either TEGMA or DMAEMA, which are randomly copolymerized with BuMA, respectively, take part in the core formation, which might not be the case.

Concerning the diblock bipolymers, Polymer 9, which is the most hydrophilic, shows the expected trend at both pH values, i.e., the experimental values are smaller than the theoretical values. Concerning Polymer 8, the expected trend is observed at pH 6 at which the difference in the hydrophobicity of BuMA and DMAEMA is more pronounced. However, at pH 7, the opposite trend is observed which can be attributed to the effect of hydrophobicity. This confirms the explanation given before about the hydrophobicity enhancing the aggregation to some extent.

The experimental value of the statistical copolymer is almost six times higher than the theoretical one corresponding to random coil configuration. This is consistent with other studies, in which bigger structures were detected by DLS [18,22]. This can be ascribed to (i) the presence of the lengthy side chains which might favor aggregation [26], and (ii) the good compatibility of TEGMA and DMAEMA with water, thus enhancing the interactions with water and favoring the extension of the polymer chain [18]. In addition, the experimental value of 15.7 nm is closer to the value of 13.5 nm which corresponds to the length of one fully extended polymer chain [(DP_TEGMA_ + DP_BuMA_ + DP_DMAEMA_) × 0.254 nm], rather than then size of the random coil which was calculated at 2.7 nm. The experimental value is slightly bigger than the length of a fully extended polymer chain. This could be attributed to the fact that the long side chains of TEGMA units have not been taken into account in the calculations. As opposed to one of our earliest studies [18], in which the size was small enough to ensure no micelle or aggregate formation, in this case, this cannot be verified by the DLS results; this is consistent with our latest study [22]. The difference may be attributed to different compositions but nevertheless, another characterization technique, specifically TEM, was carried out to provide information about the state of the statistical copolymer in solution.

#### 3.2.2. Transmission Electron Microscopy Images

**Figure 5:**
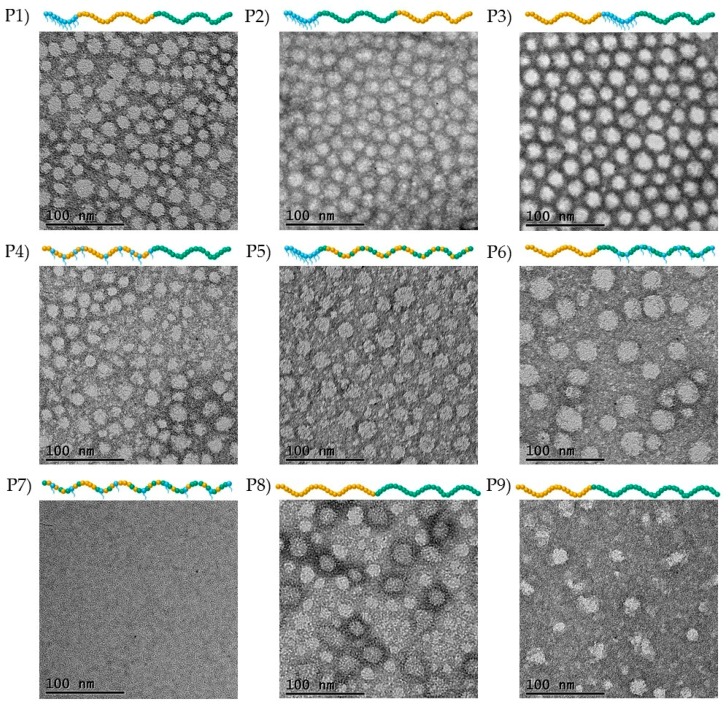
Transmission electron microscopy (TEM) images of 1% *w*/*w* negatively-stained copolymer solutions at pH 6. The scale bar is set at 100 nm. The schematics of the polymer structures are also given; Blue, orange, and green spheres represent the TEGMA, BuMA, and DMAEMA units, respectively.

To confirm the hypothesis that the block copolymers form spherical micelles and provide more details concerning the state of the statistical copolymer, TEM images were recorded, which are shown in Figure 5.

As mentioned before, the TEM images at 100 nm scale are shown in Figure 5. As a reminder the pH of the solutions used to prepare the TEM samples was adjusted to 6 to ensure that all polymers were soluble in water. From the TEM results, it can be confirmed that the block copolymers form spherical-like micelles, whereas the statistical copolymer does not. The diameter of these spherical micelles in most cases is around 20 nm, with the exception of Polymer 6, the micelles of which appear to be slightly bigger at around 25–30 nm. This is in a good agreement with the DLS data. The only significant difference is observed for Polymer 1, for which the DLS measurements showed a size of 6.5 nm. At this point, it should be remembered that the hydrodynamic diameters are determined as the size corresponding to the maximum intensity on the DLS histograms. In the case of Polymer 1, a bimodal distribution was obtained by DLS, with a second peak at higher values, which confirms the TEM results. The minor differences between the DLS and TEM results can be attributed to the different way of performing the experiments, which was previously discussed [27]. Specifically, DLS is performed in the aqueous state, in which the hydrophobic BuMA units are fully collapsed and the hydrophilic TEGMA and DMAEMA units are fully expanded. On the other hand, TEM is conducted in the dry state [27]. Therefore, it is concluded that the TEM images are not representative of the actual structure of the micelles in solution, since they are recorded in the dry state. However, they give a good approximation of the micelles size and shape, i.e., the size is close to the one obtained by DLS, and formation of spherical-like micelles is revealed by the block copolymers. It is also confirmed by TEM that no micelles are formed by the statistical copolymer, which complements the DLS results.

#### 3.2.3. Effective p*K*_a_s

**Figure 6:**
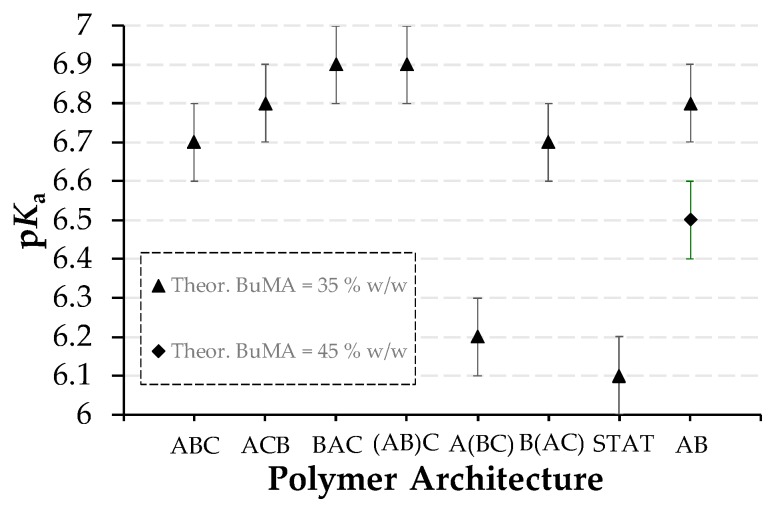
Effect of the polymer architecture and the theoretical hydrophobic BuMA weight percentage on the p*K*_a_.

The effective p*K*_a_s of the copolymers, as determined by hydrogen ion titrations, are listed in Table 2 and are shown in Figure 6. In most of the cases, the effective p*K*_a_ values vary between 6.7 and 6.9, which are similar to previously reported p*K*_a_ values on DMAEMA-based polymers [18,19,20,21,22,28,29,30]. However, the TEGMA_9_-*b*-(BuMA_20_-*co*-DMAEMA_21_) (Polymer 5), the TEGMA_9_-*co*-BuMA_20_-*co*-DMAEMA_21_ (Polymer 7), and the BuMA_26_-*b*-DMAEMA_28_ (Polymer 8) show lower effective p*K*_a_s, specifically 6.2, 6.1, and 6.5, respectively. The low p*K*_a_ of the statistical copolymer is in agreement with other studies on similar statistical terpolymers studied by our group and it is attributed to its inability to form micelles and stabilize itself in solution [18]. This was also proven in this study by TEM. Compared to the previously-studied PEGMA_6_-*co*-BuMA_18_-*co*-DMAEMA_19_, with the same target MM and composition, the TEGMA-based copolymer shows significantly lower p*K*_a_ value (6.1 versus 6.7) [22]; this is due to the more hydrophobic TEGMA unit replacing the PEGMA one. The lower p*K*_a_ of Polymer 8 is explained upon considering its higher hydrophobic BuMA content, which decreases the dielectric constant, similar to other studies [19,20,21,22,31,32]. Concerning Polymer 5, this is the first systematic study in which this type of architecture has been studied. It can be concluded that randomly copolymerizing the pH-responsive unit DMAEMA with the hydrophobic BuMA one makes the DMAEMA units weaker, based on their steric hindrance and the surrounding hydrophobic environment. Among the other copolymers, it can be observed that the more exposed the DMAEMA units are (see micelle structures in Figure 4), the easier the protonation, thus the stronger the base and the higher the p*K*_a_ values are.

#### 3.2.4. Cloud Points

Table 2 lists the CP of 1% *w*/*w* copolymer solutions in DI water when the DMAEMA units were not protonated and were protonated by 10%. At the initial pH (no protonation), only three of the solutions were homogeneous and thus they were able to be visually tested; specifically, Polymers 1, 4, and 5. The rest of the polymer solutions were insoluble. The insolubility can be ascribed to (i) their architecture (Polymers 2, 3, 6, and 7) and (ii) the increased hydrophobicity of Polymers 8 and 9 (absence of hydrophilic TEGMA and increased hydrophobic content). While Polymer 1 and 5 present a CP at 29 and 32 °C, respectively, Polymer 4 did not respond to temperature, presumably on account of its architecture, which increases the hydrophilicity; the TEGMA groups are distributed in the same block as the hydrophobic BuMA. On the other hand, when the DMAEMA units were protonated by 10%, none of the solutions presents a CP within the temperature range tested. This shows that the protonation of the DMAEMA units, even by only 10%, increases the hydrophilicity of the structure and also prevents aggregation of the micelles on account of the electrostatic repulsion between the positively charged amino groups. The effect of protonation on the thermoresponse of DMAEMA units is well-documented [33,34].

#### 3.2.5. Visual Gel Points

**Figure 7:**
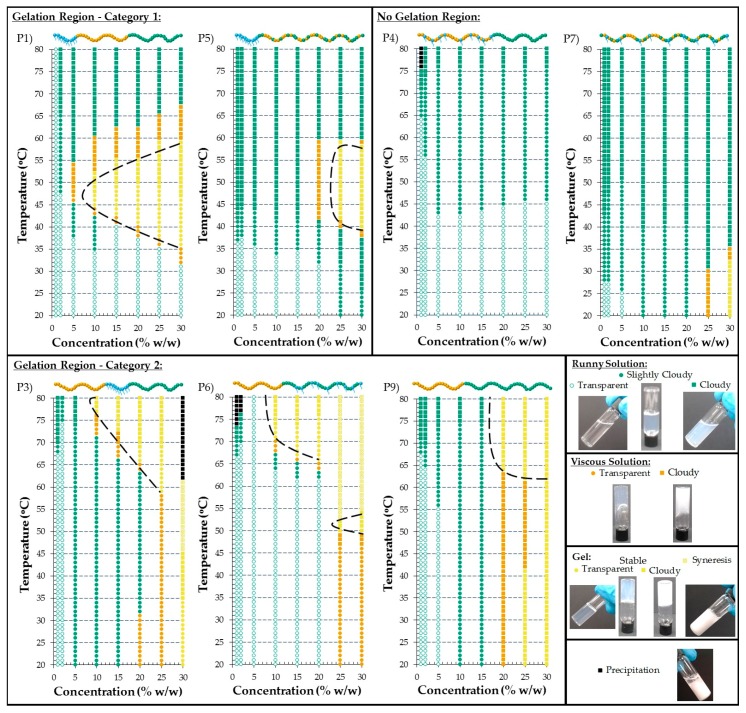
Phase diagrams of the TEGMA-based copolymer solutions in phosphate buffered saline (PBS) of various architectures, namely ABC (Polymer 1), BAC (Polymer 3), (AB)C (Polymer 4), A(BC) (Polymer 5), B(AC) (Polymer 6), and the statistical terpolymer (Polymer 7). The phase diagram of the diblock bipolymer BuMA_20_-*b*-DMAEMA_34_ (Polymer 9) is also shown. The concentrated copolymer solutions in PBS of the ACB (Polymer 2), and the most hydrophobic diblock bipolymer (Polymer 8) were not soluble, thus their phase diagrams are not presented. The transparent and slightly cloudy runny solutions are presented in white and green circles, respectively, whereas the cloudy ones are indicated by green squares. The transparent and cloudy viscous solutions are shown in orange circles and squares, respectively. The transparent and cloudy stable gels are indicated by yellow circles and squares, respectively. Gel syneresis is represented by white squares with diagonal yellow lines, while the precipitation is shown in black squares. The gelation region is approximately shown with the black dashed line. The corresponding polymer structures are schematically given, in which the TEGMA, BuMA, and DMAEMA units are colored in blue, orange, and green, respectively.

Phase diagrams were constructed for seven out of nine polymers. Specifically, the diluted and concentrated solutions of the following polymers in PBS formed homogeneous mixtures (either solutions or gels) and they were visually tested for thermoresponse. The phase diagrams of TEGMA_9_-*b*-BuMA_20_-*b*-DMAEMA_21_ (Polymer 1), BuMA_20_-*b*-TEGMA_9_-*b*-DMAEMA_21_ (Polymer 3), (TEGMA_9_-*co*-BuMA_20_)-*b*-DMAEMA_21_ (Polymer 4), TEGMA_9_-*b*-(BuMA_20_-*co*-DMAEMA_21_) (Polymer 5), BuMA_20_-*b*-(TEGMA_9_-*co*-DMAEMA_21_) (Polymer 6), TEGMA_9_-*co*-BuMA_20_-*co*-DMAEMA_21_ (Polymer 7), and BuMA_20_-*b*-DMAEMA_34_ (Polymer 9) are shown in Figure 7. The other two polymers, TEGMA_9_-*b*-DMAEMA_21_-*b*-BuMA_20_ (Polymer 2) and BuMA_26_-*b*-DMAEMA_28_ (Polymer 8), were only soluble at the lowest concentrations and therefore, the construction of meaningful phase diagrams was not feasible.

All the copolymer solutions at 1% *w*/*w* showed a CP within the temperature range tested, with the exception of Polymer 1. The non-thermoresponse of Polymer 1 can be attributed to the significantly smaller experimental hydrodynamic diameter of the micelles formed by Polymer 1; its size is less than half of the size of the micelles formed by the other polymers. While all the copolymer solutions showed thermoresponse at 70 °C and above, the solution of Polymer 5 interestingly showed thermoresponse at 42 °C. This demonstrates an architectural effect which, by randomly copolymerizing the thermoresponsive unit DMAEMA with the hydrophobic BuMA, while keeping the hydrophilic TEGMA unit as a distinct block, enhances the thermoresponse. This can be explained by considering the enhanced hydrophobic environment surrounding the thermoresponsive unit. This is in contrast with the results obtained by testing the solutions in DI water, during which no thermoresponse was observed. This is due to the ionic strength effect, i.e., screening of the electrostatic repulsions between the protonated DMAEMA units. The ionic strength effect on the LCST of DMAEMA units has been previously observed and discussed by De Souza et al. [33].

Since physical gels have interesting applications, including injectable gels and 3-D printing, the solutions were visually tested for gelation. The gelation region (if there is one), i.e., temperature and concentration ranges where a stable gel was formed, is approximately shown by the black dashed line in Figure 7. The points which correspond to stable transparent and cloudy gel are shown in yellow circles and squares, respectively. Interestingly, a gelation region was observed for five out of six polymers. Specifically, Polymers 1, 3, 5, 6, and 9, formed a stable gel, whereas the solutions of Polymers 4 and 7 only presented a CP, regardless of the concentration of the solution; the formation of a stable gel by the most concentrated solution of the statistical terpolymer can be attributed to reaching the limit of entanglements. The inability of Polymer 4 to form a gel, even in highly concentrated solutions, demonstrates, once again, an architectural effect. Particularly, by randomly copolymerizing the hydrophobic BuMA unit with the hydrophilic TEGMA unit, while polymerizing the thermoresponsive DMAEMA in a distinct block, enhances the hydrophilicity of the structure, thus interrupting gelation.

The phase diagrams which include a gelation region can be divided into two categories, which differ in the thermoresponsive behavior; as is shown in Figure 7. In the first category, two copolymers are included, specifically Polymers 1 and 5, whereas Polymers 3, 6, and 9 belong to the second category. When copolymer solutions of the first category are concerned, runny solutions are formed at low temperatures, regardless of the increased concentrations. These solutions form a stable gel upon increasing the temperature, which then gets destabilized and turns back to solution when the temperature increases further. The ones belonging to the second category, show gelation at higher temperature ranges and the gels formed mostly remain stable until the highest temperature tested. The gelation temperature decreases by increasing the concentration of the solution; as expected. It is worth-noting that in these phase diagrams, the highly concentrated solutions formed either a stable gel or a highly viscous at room temperature. When a stable gel at room temperature was formed, these gels either remained stable upon heating or gel syneresis was observed at higher temperatures. In the case of highly viscous solution, a stable gel is formed upon increasing the temperature, followed by gel syneresis. The gelation at room temperature by highly concentrated polymer solutions can be ascribed to reaching the limit of entanglements rather than to thermoresponse. An interesting feature is that a clear region of transparent gel is not obtained by Polymer 9, which does not contain the hydrophilic TEGMA units and by Polymer 5, in which the thermoresponsive unit is within a hydrophobic BuMA-based environment. Regarding the rest of the polymers which formed a gel (Polymers 1, 3, and 6), a transparent gel was formed which was then transformed into a cloudy one at higher concentrations; this has been previously observed for triblock copolymers containing similar monomers [22].

Therefore, in this study, the effect of architecture on the thermoresponsive behavior is clearly demonstrated. Comparing the behavior of the three triblock terpolymers, the ABC architecture shows clear sol-gel transition, while the ACB one is insoluble and the BAC one shows gelation at higher temperatures, thus confirming the trends previously observed [18,20]. Compared to our previous study in which no gelation was observed for the ACB architecture, the gelation in the present study can be attributed to the presence of TEGMA units, instead of PEGMA units (around five repeat EG groups), which increases the hydrophobicity of the structure, thus enhancing the thermoresponse. Concerning the three diblock terpolymers, which have been systematically studied for the first time, the A(BC) architecture shows clear sol-gel transition, whereas the (AB)C one does not form gel, and the B(AC) one forms gel at higher temperatures. On the other hand, no gelation was observed for the statistical terpolymer, which is consistent with our previous studies [18,20]. Interestingly, gelation is observed at high temperatures by the AB diblock copolymer which did not show solubility issues, specifically Polymer 9, as opposed to the solubility issues of Polymer 8. In conclusion, two architectures, namely ABC with the hydrophobic BuMA forming the central block and A(BC) in which the thermoresponsive DMAEMA has been randomly copolymerized with the hydrophobic BuMA, show clear sol-gel-sol transition close to the desirable, body temperature values. Among the two, the ABC architecture is most promising since it shows wider gelation region, i.e., a stable gel is formed at lower temperatures and concentration and this gel gets destabilized at higher temperatures.

## 4. Conclusions

In this study, seven well-defined terpolymers of different architectures were successfully synthesized via GTP. The hydrophilic TEGMA, the hydrophobic BuMA, and the thermoresponsive DMAEMA were used as the A, B, and C units, respectively. The architecture of the polymers was systematically varied in order to investigate its effect on the thermoresponsive behavior of the copolymers. Three architectures, namely ABC, ACB, BAC, and statistical, that have been previously studied, as well as three novel architectures, specifically (AB)C, A(BC), and B(AC) were investigated and compared. Two BC diblock bipolymers mimicking the BuMA:DMAEMA and BuMA:(TEGMA + DMAEMA) ratios of the weight percentages in the terpolymers were also synthesized. All block copolymers formed spherical micelles unlike the statistical copolymer. Interestingly, the block architecture and the position of the hydrophobic groups strongly influence the thermoresponsive behavior with the ABC and A(BC) architectures to show the desirable, clear sol-gel transition close to body temperature.

## Figures and Tables

**Table 1:** Theoretical and experimental molar masses (MMs) and compositions, and molar mass distributions (MMDs) of the copolymers and their precursors.

Polymer No.	Theoretical polymer structure ^a^	% *w*/*w* TEGMA-BuMA-DMAEMA		MM Theor. ^b^ (g·mol^−1^)	*M*_n_^c^ (g·mol^−1^)	*Ð* ^c^
		Theoretical	^1^H-NMR			
1	TEGMA_9_	100-00-00	100-00-00	2125	3020	1.12
	TEGMA_9_-*b*-BuMA_20_	42-58-00	42-58-00	4960	6600	1.12
	TEGMA_9_-*b*-BuMA_20_-*b*-DMAEMA_21_	25-35-40	25-35-40	8200	10,700	1.17
2	TEGMA_9_	100-00-00	100-00-00	2125	3030	1.11
	TEGMA_9_-*b*-DMAEMA_21_	38-00-62	38-00-62	5365	7650	1.11
	TEGMA_9_-*b*-DMAEMA_21_-*b*-BuMA_20_	25-35-40	26-34-40	8200	10,800	1.16
3	BuMA_20_	00-100-00	00-100-00	2935	2980	1.12
	BuMA_20_-*b*-TEGMA_9_	42-58-00	41-59-00	4960	6700	1.08
	BuMA_20_-*b*-TEGMA_9_-*b*-DMAEMA_21_	25-35-40	26-35-39	8200	10,100	1.13
4	TEGMA_9_-*co*-BuMA_20_	42-58-00	42-58-00	4960	5940	1.09
	(TEGMA_9_-*co*-BuMA_20_)-*b*-DMAEMA_21_	25-35-40	26-35-39	8200	8780	1.10
5	TEGMA_9_	100-00-00	100-00-00	2125	2810	1.12
	TEGMA_9_-*b*-(BuMA_20_-*co*-DMAEMA_21_)	25-35-40	26-34-40	8200	9950	1.14
6	BuMA_20_	00-100-00	00-100-00	2935	4180	1.10
	BuMA_20_-*b*-(TEGMA_9_-*co*-DMAEMA_21_)	25-35-40	26-37-37	8200	8580	1.13
7	TEGMA_9_-*co*-BuMA_20_-*co*-DMAEMA_21_	25-35-40	26-35-39	8200	8760	1.10
8	BuMA_26_	00-100-00	00-100-00	3826	6240	1.07
	BuMA_26_-*b*-DMAEMA_28_	00-46-54	00-47-53	8200	11,000	1.11
9	BuMA_20_	00-100-00	00-100-00	2935	4040	1.10
	BuMA_20_-*b*-DMAEMA_34_	00-35-65	00-37-63	8200	9380	1.10

^a^ TEGMA, BuMA, and DMAEMA stand for tri(ethylene glycol) methyl ether methacrylate, *n*-butyl methacrylate, and 2-(dimethylamino) ethyl methacrylate, respectively; ^b^ The theoretical MM of the polymer was calculated as follows: (DP_TEGMA_ × MM_TEGMA_) + (DP_BuMA_ × MM_BuMA_) + (DP_DMAEMA_ + MM_DMAEMA_) + 100 g·mol^−1^; MM and DP stand for molar mass and degree of polymerization, respectively. The value of 100 g·mol^−1^ is the part of the MTS initiator which stays on the polymer chain; ^c^ The *M*_n_ and *Ð* were determined by gel permeation chromatography (GPC). The calibration curve was plotted using six linear poly (methyl methacrylate) (pMMA) standard samples of MM equal to 2000, 4000, 8000, 20,000, 50,000, and 100,000 g·mol^−1^.

**Table 2:** Theoretical and experimental hydrodynamic diameters, effective p*K*_a_s and cloud points of 1% *w*/*w* aqueous copolymer solutions.

Polymer No.	Theoretical polymer structure ^a^	Hydrodynamic diameter (nm)			Effective p*K*_a_s ± 0.1	Cloud Points ± 2 °C	
		Theoretical ^b^	Experimental ± 0.5			0% H^+^	10% H^+^
			pH = 7	pH = 6			
1	TEGMA_9_-*b*-BuMA_20_-*b*-DMAEMA_21_	20.5 ^c^	8.7	6.5	6.7	29	No CP
2	TEGMA_9_-*b*-DMAEMA_21_-*b*-BuMA_20_	26.6 ^d^	32.7	32.7	6.8	NA	No CP
3	BuMA_20_-*b*-TEGMA_9_-*b*-DMAEMA_21_	24.8 ^d^	18.2	13.5	6.9	NA	No CP
4	(TEGMA_9_-*co*-BuMA_20_)-*b*-DMAEMA_21_	19.1 ^e^	21.0	24.4	6.9	No CP	No CP
5	TEGMA_9_-*b*-(BuMA_20_-*co*-DMAEMA_21_)	18.1 ^f^	25.5	21.0	6.2	32	No CP
6	BuMA_20_-*b*-(TEGMA_9_-*co*-DMAEMA_21_)	20.8 ^d^	28.2	18.2	6.7	NA	No CP
7	TEGMA_9_-*co*-BuMA_20_-*co*-DMAEMA_21_	2.7 ^g^	NA	15.7	6.1	NA	NA
8	BuMA_26_-*b*-DMAEMA_28_	28.1 ^c^	32.7	15.7	6.5	NA	No CP
9	BuMA_20_-*b*-DMAEMA_34_	25.3 ^c^	21.0	15.7	6.8	NA	No CP

^a^ TEGMA, BuMA, and DMAEMA stand for tri(ethylene glycol) methyl ether methacrylate, *n*-butyl methacrylate, and 2-(dimethylamino) ethyl methacrylate, respectively; ^b^ The theoretical hydrodynamic diameters (*d*_h_) were calculated by taking into account the experimental degrees of polymerization (DP) as resulted from GPC and ^1^H-NMR analysis; ^c^ The theoretical values of the ABC triblock terpolymer and the BC diblock bipolymers were calculated based on the following equation: *d*_h_ = (DP_BuMA_ + 2 × DP_DMAEMA_) × 0.254 nm; ^d^ For the calculation of the theoretical hydrodynamic diameters of the ACB and BAC triblock and the B(AC) diblock terpolymers, the following equation was used: *d*_h_ = [DP_BuMA_ + 2 × (DP_DMAEMA_ + DP_TEGMA_)] × 0.254 nm; ^e^ The following formula was used in the case of the (AB)C diblock terpolymer: *d*_h_ = (DP_BuMA_ + DP_TEGMA_ + 2 × DP_DMAEMA_) × 0.254 nm; ^f^ In the case of the A(BC) diblock terpolymer, the theoretical value was calculated according to the following equation: *d*_h_ = (DP_BuMA_ + DP_DMAEMA_ + 2 × DP_TEGMA_) × 0.254 nm; ^g^ A random coil configuration was assumed to be adopted by the statistical copolymer and therefore the equation for a random coil was used: <*d*_g_^2^>^1/2^ = 2 × [2 × 2.20 × (DP_TEGMA_ + DP_BuMA_ + DP_DMAEMA_)/3]^1/2^ × 0.154 nm; NA Testing these copolymer solutions was not feasible because the copolymers were insoluble at this pH.

## Supplementary Materials

The following are available online at www.mdpi.com/2073-4360/9/1/31/s1. Figure S1 shows the GPC chromatograms of the triblock terpolymers (Polymers 1–3), the diblock terpolymers (Polymers 4–6), the statistical terpolymer (Polymer 7), the diblock bipolymers (Polymers 8 and 9), and their linear precursors. The GPC traces of the first block and the diblock are indicated by blue solid and orange dotted lines, whereas the final triblock terpolymer (if there is any) is shown by green solid line. The GPC chromatogram of the statistical terpolymer is colored in black. Green dashed line represents the GPC traces of the final terpolymers after precipitation; Figure S2 shows the ^1^H NMR spectra of Polymer 1 before precipitation and its linear precursors: (a) TEGMA_9_, (b) TEGMA_9_-*b*-BuMA_20_, and (c) TEGMA_9_-*b*-BuMA_20_-*b*-DMAEMA_21_ are colored in blue, orange, and green, respectively.
