# Supplementary material for: A Comprehensive Systematic Study on Thermoresponsive Gels: Beyond the Common Architectures of Linear Terpolymers
Source: Polymers (Basel). 2017 Jan 20;9(1):31. doi: 10.3390/polym9010031 (PMC6432086; doi:10.3390/polym9010031)
Supplement: Supplementary file 1 [file polymers-09-00031-s001.pdf]

Supplementary Materials: A Comprehensive Systematic Study on Thermoresponsive Gels: Beyond the Common Architectures of Linear Terpolymers

Anna P. Constantinou, Hanyi Zhao, Catriona M. McGilvery, Alexandra E. Porter and Theoni K. Georgiou

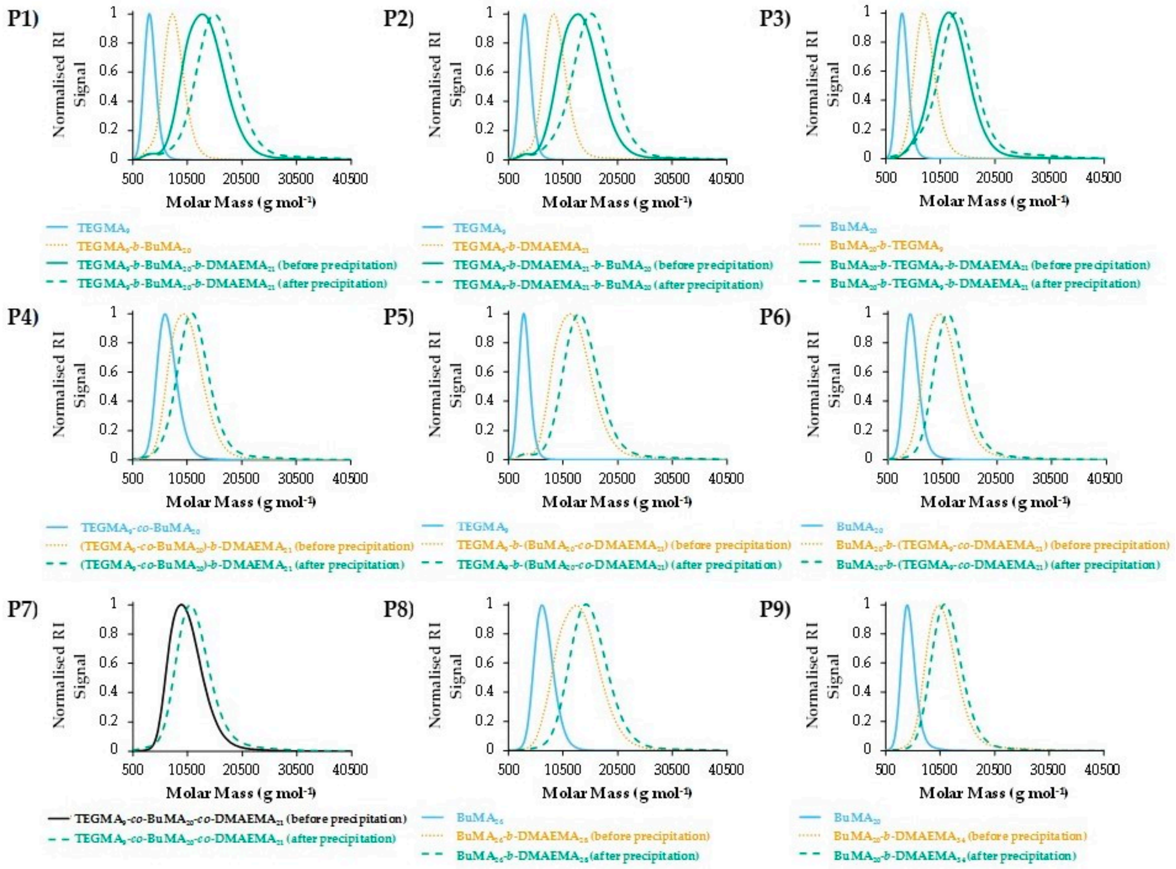

Figure S1. Gel permeation chromatography (GPC) chromatograms which confirm the synthesis of the triblock terpolymers (Polymers 1–3), the diblock terpolymers (Polymers 4–6), the statistical terpolymer (Polymer 7), and the diblock bipolymers (Polymers 8 and 9). The GPC traces of the first block, the diblock, and the triblock (if there is one) copolymers are shown by blue solid, orange dotted, and green solid lines, respectively. The GPC trace of the statistical terpolymer is colored in black. The GPC chromatograms of the final copolymers after precipitation are given by a green dashed line.

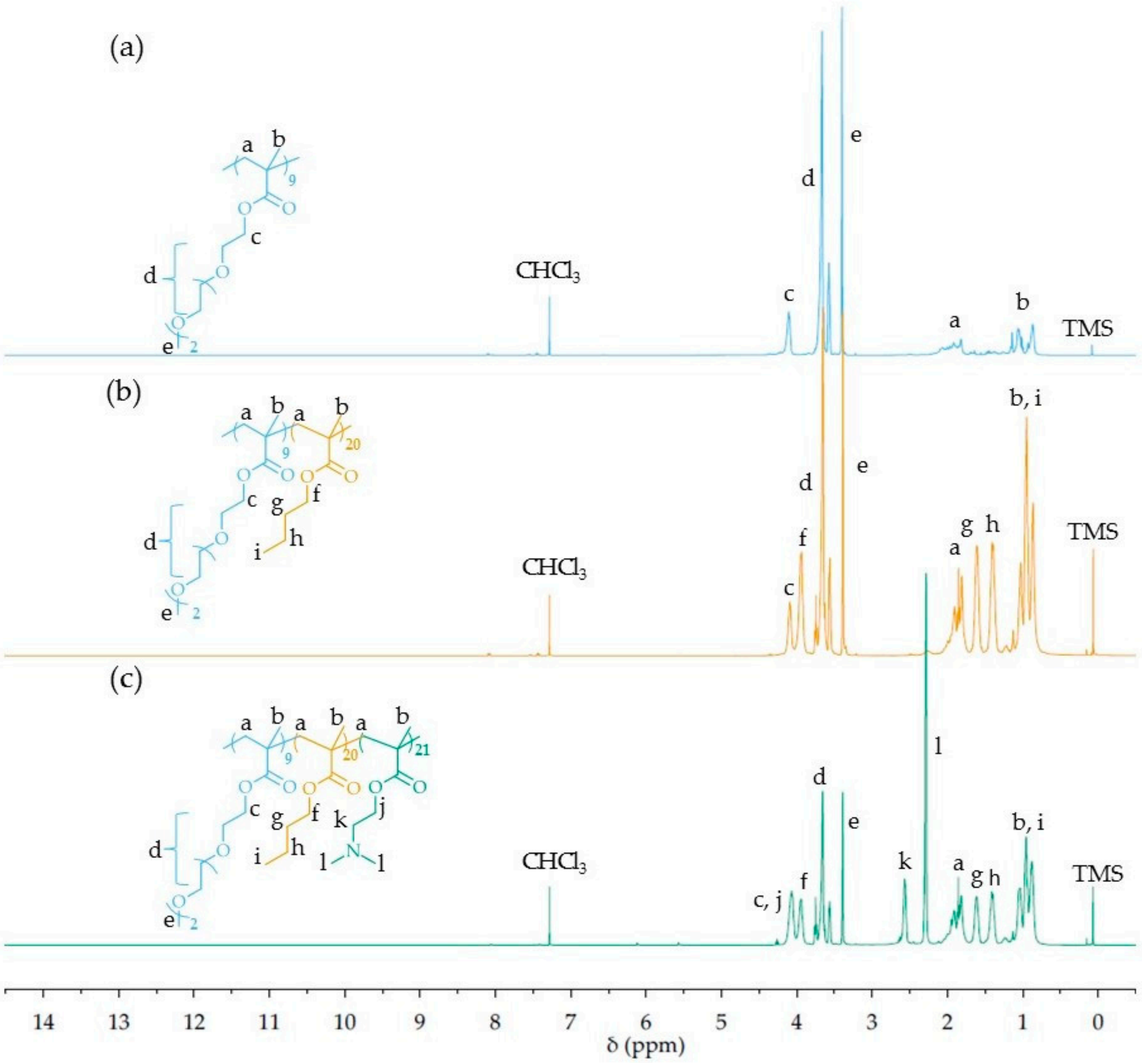

Figure S2. ^{1}H-NMR spectra of Polymer 1 ($TEGMA_9$-*b*-$BuMA_{20}$-*b*-$DMAEMA_{21}$) and its precursors. The spectra of (**a**) the TEGMA homopolymer ($TEGMA_9$); (**b**) the diblock ($TEGMA_9$-*b*-$BuMA_{20}$); and (**c**) the triblock copolymer before precipitation are colored in blue, orange, and green, respectively.
